# An Early Presentation of Tricuspid Valve Rupture in a Trauma Patient With Congenital Heart Disease

**DOI:** 10.1155/cris/6711702

**Published:** 2025-01-30

**Authors:** Justus Boever, Rishi Batra, Hason Khan, Zachary M. Bauman

**Affiliations:** Division of Trauma, Emergency General Surgery and Critical Care Surgery, Department of Surgery, University of Nebraska Medical Center, Omaha, Nebraska, USA

## Abstract

Tricuspid valve regurgitation/rupture is a rare complication of trauma, with only around 150 cases reported in the literature, though this prevalence may be underestimated due to subtle clinical manifestations. The tricuspid valve is the most frequently affected heart valve following blunt chest trauma due to its anterior anatomical position between the sternum and the vertebrae. The diagnosis of tricuspid regurgitation is often delayed in the traumatic setting due to the subtlety of clinical manifestations. Many trauma patients also present with distracting injuries. The subsequent treatment delay can result in development of irreversible dilatation of right-sided heart chambers, making it imperative to have a high index of suspicion for tricuspid regurgitation as a cause of acute hemodynamic instability in the setting of blunt trauma to the chest. In this report, we present a unique case of traumatic tricuspid valve regurgitation in a patient with a history of congenital atrial septal defect (ASD)/partial anomalous pulmonary venous return (PAPVR).

## 1. Introduction

Partial anomalous pulmonary venous return (PAPVR) is defined as a left-to-right shunt involving one or more, but not all, pulmonary veins draining into the right atrium [1]. PAPVR that involves the right upper and/or the middle pulmonary vein can be associated with a sinus venosus atrial septal defect (ASD), in which an abnormally inserted superior or inferior vena cava overrides the interatrial septum [2]. Sinus venosus ASDs account for 10% of atrial communications, though they do not represent true ASDs, since the interatrial communication lies within the sinus venosus septum rather than the embryologic atrial septum [2, 3]. Potential complications of untreated ASDs in adults include arrhythmias, paradoxical embolization, and right ventricular failure, as well as pulmonary hypertension which can lead to right-to-left shunting (Eisenmenger syndrome) [3–5]. Sinus venosus ASDs are usually diagnosed using echocardiography, but computed tomography (CT) can also be used [4, 5]. ASD closure is indicated in patients with functional impairment with hemodynamically significant left-to-right shunting without significant pulmonary hypertension [4, 5]. Surgical closure is the standard management, though transcatheter closure through superior vena cava stent insertion with or without device implantation has recently been shown as a feasible and effective alternative [6].

Tricuspid regurgitation is defined as backflow of blood during systole from the right ventricle into the right atrium. Overall, only 10% of cases of severe tricuspid regurgitation are primary, which is due to processes directly affecting the tricuspid valve rather than to secondary conditions such as left-sided heart failure [7]. Tricuspid valve regurgitation/rupture is a rare complication of trauma, with only around 150 cases reported in the literature, though this prevalence may be underestimated due to subtle clinical manifestations [2, 8]. The tricuspid valve is the most frequently affected heart valve following blunt chest trauma due to its anterior anatomical position between the sternum and the vertebrae [3]. The most common cause of traumatic tricuspid regurgitation/rupture is abrupt deceleration coupled with increased right-sided heart pressures (due to Valsalva maneuver and/or thorax compression) [9]. Mechanisms of injury typically involve the subvalvular apparatus including anterior and posterior chordal or papillary muscle rupture versus a laceration of leaflet combined with chordal rupture [10]. Patients may become symptomatic immediately or experience delayed presentation due to mechanical fatigue [11, 12]. Continued regurgitation through the compromised valve over time results in annular dilation and right ventricular enlargement [6]. When severe, chronically elevated right heart pressures and effective volume overload can lead to right ventricular systolic dysfunction, low forward cardiac output, and signs and symptoms of right heart failure [6]. Treatment options include surgical valve repair or replacement versus long-term medical therapy and observation [8, 13]. In this case report, we present a delayed presentation of traumatic tricuspid valve regurgitation in patient with congenital cardiac disease.

## 2. Case Report

A 69-year-old male with a significant cardiac history of congenital ASD/PAPVR repair as a child, atrial fibrillation on chronic anticoagulation and congestive heart failure with left ventricular dysfunction, presented as limited activation following a motor vehicle crash (MVC) as a restrained passenger. In the trauma bay, the patient demonstrated left-sided chest pain and was found to have fractures of his left ribs 5–8, a fracture of the left tibial plateau, and a left ulnar fracture. Due to significant left chest pain, the severe displacement of his left rib fractures, worsening respiratory status and the fact he was feeling these rib fractures “clicking” with respirations, open reduction and internal fixation (ORIF) of the left rib fractures was recommended.

On hospital day 2, the patient was taken to the operating room for ORIF of his left rib fractures 5–8, after completion of his cardiac risk stratification which included an echocardiogram showing stable cardiac function. Of note, 2 months prior to the patient's admission, he underwent elective hip arthroplasty without any surgical or cardiac complications. Upon induction, however, the patient became acutely hypoxic and was thought to have developed a left tension pneumothorax. Immediate needle decompression followed by chest tube placement was performed, draining 300 ml of hemorrhagic fluid. However, the patient's clinical status remained unchanged. Emergent bronchoscopy followed, resulting in removal of bilateral mucous plugging, but this too did not relieve the hypoxia. Finally, intraoperative transesophageal echocardiogram (TEE) was performed, revealing severe tricuspid regurgitation and significant right-to-left shunting secondary to what appeared to be a worsening sinus venosus defect coupled with the likely new diagnosis of tricuspid valve rupture. The procedure was subsequently aborted and a variety of techniques were utilized to reverse the right-to-left shunt. This included initiation of epoprostenol as well as phenylephrine. Furthermore, the patient was extubated with a pulse oximetry of only 82% due to the increased intrathoracic pressure worsening the shunt. Upon implementing these techniques, we were able to bring the patient's oxygen saturation up to 95%. The patient was transferred to the intensive care unit (ICU) for continued care and close monitoring.

**Figure 1:**
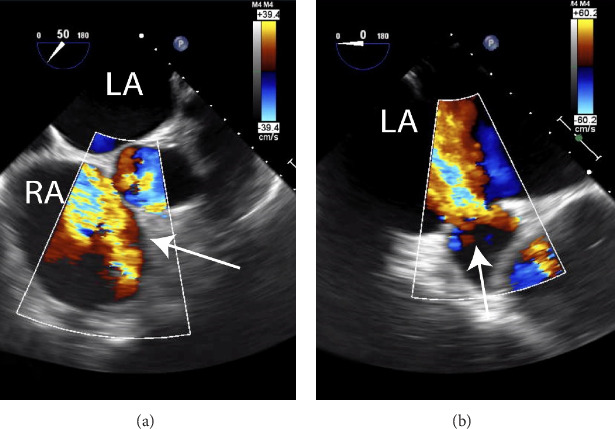
(A) Transesophageal echocardiogram (TEE) showing the mid-esophageal modified right ventricular inflow view. There is severe tricuspid regurgitation (marked with arrow). (B) TEE showing a lower esophageal view of the inferior sinus venosus atrial septal defect (ASD). This image shows a high-velocity jet with right-to-left shunting. This is the result of the traumatic severe TR and the jet is directed at the sinus venosus ASD causing the right-to-left shunting (measuring 75 and <10 mm^2^).

A formal cardiology consultation was obtained, and the intraoperative TEE was reviewed. A newly recognized flailed anterior leaflet of tricuspid valve was confirmed which, during induction for the attempted rib fixation procedure, directed regurgitant jets through two residual ASDs that had been present ever since his patch repair for sinus venosus ASD as a child. Clinically, the patient remained in the ICU and developed impressive jugular venous distension from the traumatic tricuspid regurgitation. He continued to require oxygen support with oxygen saturations in the low 90′s. Repeat TEE confirmed flailed anterior leaflet of tricuspid valve with severe regurgitation. Regurgitant jet shunting of blood continued to be noted flowing from right to left across residual ASD (Figure 1A). Furthermore, the degree of shunting did not change significantly upon withdrawal of prostacyclin infusion. Two residual ASDs were identified at site of prior sinus venosus repair measuring 75 and <10 mm^2^ on 3-deminsional imaging (Figure 1B). Cardiothoracic surgery consultation was recommended for evaluation of tricuspid valve repair. Overall, this resulted in a dilated right ventricle with mildly depressed ejection fraction (EF; 40%–45%) and a patient unfit for surgical repair of his ribs or orthopedic fractures. Preoperative cardiac catheterization was performed ruling out any coronary artery disease. The patient was subsequently taken for surgery by CT surgery given the severe tricuspid regurgitation and worsening of the sinus venosus ASD. Intraoperatively, he was found to have severe pericardial adhesions from his prior operation. An inferior dehiscence of prior patch closure of ASD was found in addition to severe flail of the anterior leaflet of the tricuspid value. The ASD was closed with a pericardial patch and the injured tricuspid valve was replaced with a 33 mm bioprosthesis valve. The remainder of his postoperative course was uneventful and the patient was discharged to skilled nursing facility on POD#7/hospital day 18.

## 3. Discussion

In this report, we present a unique case of traumatic tricuspid valve regurgitation in a patient with a history of congenital ASD/PAPVR. Several options exist for treatment of hypoxia in the setting of a newly discovered right-to-left shunt. Intravenous infusion of prostaglandins such as PGI2, or epoprostenol reduces pulmonary artery pressure and pulmonary vascular resistance due to its long-lasting vasodilatory effect, thereby, decreasing right heart pressures and increasing forward cardiac output [14]. Vasopressors help achieve the goal of maintaining preload, as do interventions to decrease intrathoracic pressure such as extubation as early as possible [15].

The diagnosis of tricuspid regurgitation is often delayed in the traumatic setting due to the subtlety of clinical manifestations. Many trauma patients also present with distracting injuries [8, 11, 12, 16, 17]. The subsequent treatment delay can result in development of irreversible dilatation of right-sided heart chambers [8]. Therefore, it is imperative to maintain a high index of suspicion for tricuspid regurgitation as a cause of acute hemodynamic instability in the setting of blunt trauma to the chest, especially with sudden deceleration mechanisms and in patients with a congenital cardiac history [17]. In one study of 10 patients with traumatic tricuspid regurgitation, the mean interval between initial trauma and diagnosis was 74.1 months and the mean interval between trauma and surgical intervention was 81.8 months [13]. The use of a combination of transthoracic echocardiography and magnetic resonance imaging provides the best volumetric and contractile assessment of the right ventricle. Multimodal imaging (e.g., real-time 3-dimensional echocardiogram) helps determine optimal timing and procedure of surgery in asymptomatic patients by characterizing morphology and function of the valve [9, 18].

In the case of this patient, the discovery of severe traumatic tricuspid regurgitation and the need for subsequent repair prevented surgical intervention for his other trauma-associated injuries, namely, rib fixation for consecutive rib fractures and repair of the left tibial plateau fracture. Instead, these injuries were monitored closely for proper healing, which likely led to a more prolonged recovery period than if surgical intervention had been feasible. For instance, in patients with three or more displaced rib fractures, prompt chest wall stabilization via surgical rib fixation has been shown to improve multiple outcomes when compared to conventional conservative management. These include decreased incidence of pneumonia, shortened length of ICU and hospital stay, decreased use of narcotics, earlier mobilization, and more rapid return to work [19–29].

## Data Availability

No raw data were utilized for this project.
